# Evaluating the Feasibility of Web-Monitoring Methodology for Measuring Exposure to Online Cancer Misinformation

**DOI:** 10.2196/65887

**Published:** 2025-07-29

**Authors:** Cindy A Turner, Andy J King, Ida Tovar, Morgan M Millar, Rachel R Codden, Jia-Wen Guo, Skyler Johnson, Anne C Kirchhoff, Margaret Raber, Xiaoming Sheng, Deanna Kepka, Echo L Warner

**Affiliations:** 1 Cancer Control and Population Sciences, Huntsman Cancer Institute, 2000 Cir of Hope Dr, Salt Lake City, UT, 84112, United States, 1 8015877000; 2 College of Nursing, University of Utah, Salt Lake City, UT, United States; 3 Department of Communication, University of Utah, Salt Lake City, UT, United States; 4 Division of Epidemiology, University of Utah, Salt Lake City, UT, United States; 5 Department of Radiation Oncology, Huntsman Cancer Institute, Salt Lake City, UT, United States; 6 MD Anderson Cancer Center, University of Texas, Houston, TX, United States

**Keywords:** online communication, cancer, misinformation, social media, social support, information, support, web monitoring, communication, cancer misinformation, acceptability, pilot study, web content, surveys, mobile device, online platform

## Abstract

Understanding the impact of online cancer misinformation exposure on health outcomes is an area of growing concern, but few methods exist to objectively measure this exposure. The primary aim of this paper is to describe the lessons learned in using web-monitoring software to measure exposure to online cancer misinformation among patients with cancer. These lessons learned emerged from our experience conducting a prospective pilot study from October 2022 to August 2023 wherein we adopted commercially available web-monitoring software to capture cancer-related web content. A total of 56 patients with cancer completed a baseline survey, and 17 of these participants installed web-monitoring software on their personal computer for 30 days and completed a follow-up survey. We use implementation outcomes to describe the feasibility of this methodological approach using lessons learned in 3 topic areas, namely data quality, software implementation, and participant acceptability. We found the web-monitoring data to be appropriate for our research aim to objectively measure cancer misinformation exposure, although compatibility issues with social media websites and mobile devices negatively impacted data quality. A complex installation process negatively impacted implementation and caused an unknown number of participants to drop out after the baseline survey. Among participants who completed the study, reported acceptability of web-monitoring software for research purposes was high, though potentially biased by selective retention. This pilot study testing web-monitoring software for research purposes among patients with cancer demonstrates high acceptability but low feasibility due to implementation barriers. We propose practical solutions to address these barriers and believe the lessons learned here offer a promising foundation for improving methods to objectively measure patient exposure to online cancer information. Future studies should focus on exploring perceptions of web-monitoring among nonparticipants, considering alternative approaches, and expanding web-monitoring to include mobile devices.

## Overview

Health information and social support are increasingly sought online, with nationally representative surveys indicating 83% of US adults use social media and 70% search for health information online [1,2]. Patients with cancer use digital resources even more often for health-related reasons—94% of patients with cancer report going online in search of cancer information [3]. While the internet can be a positive, high-quality, and easily accessible source of information and support, there is growing concern over the amount of online health misinformation, that is, information that is misleading, false, or inaccurate, which tends to spread more quickly than accurate information [4,5].

Online misinformation is particularly challenging to navigate in the context of cancer, a highly complex and heterogeneous group of diseases that are also extremely dynamic due to quickly evolving treatments. Exacerbating these complexities are demonstrated financial incentives for many cancer influencers that exist for promoting unproven miracle cures and solutions for symptom management, as well as conspiracies about the causes of cancer [6]. Cancer misinformation can negatively impact health outcomes and increase mortality risk if patients forgo conventional cancer treatment for unproven alternatives [7,8]. These risks demonstrate an urgency to strengthen the growing field of online cancer misinformation scholarship.

A recent report highlighted 2 major areas of need within online cancer misinformation research: (1) enhancing surveillance to better understand the characteristics of misinformation messages and how they spread and (2) assessing associated health outcomes [9]. The latter cannot be achieved before the former, marking a critical need for accurate and objective documentation of online cancer misinformation exposure. Most cancer misinformation studies are based on self-report, with over half of participants in a recent survey reporting misinformation on social media [10]. While studies like this are an important step toward establishing the prevalence of misinformation exposure, self-reports are notably unreliable and underestimated, in part due to differences in active (eg, posting and interacting) versus passive (eg, viewing and scrolling) online activity [9,11]. One analysis comparing 9 independent studies measuring online cancer misinformation exposure across various social media platforms found a prevalence range of 11%‐100%; such a large variation could be explained by heterogeneity in social media platforms and cancer types, or by a lack of unified methods and definitions around misinformation measurement among researchers [12]. Thus, there is a critical need to develop reliable methods that consistently collect high-quality data on cancer misinformation exposure to satisfy the demand for enhancing surveillance, spread, and impact of online health misinformation.

## Rationale for Digital Trace Data in Cancer Research

One potential avenue for addressing this gap in surveillance is to leverage digital trace data, which broadly refers to records of activity in an online information system, and can include individual metadata from social media platforms, websites, and smartphone apps [13]. To date, a few studies have integrated third-party digital trace data and survey research to answer questions about misinformation exposure; however, to our knowledge, no previous attempts have been made to integrate commercially available web-monitoring software with a prospective study design to focus specifically on online cancer-misinformation exposure [14].

Web-monitoring software is a tool to collect real-time digital trace data on an end user’s computer usage. This software is typically silently installed on the end user’s computer where it can collect, store, and analyze all activities. Examples of common web-monitoring data include time tracking (eg, when the end user is actively using their device), website and application monitoring (eg, what websites or applications the end user visits and how much time they spend there), and screen captures or recording (eg, screen captures of end user’s screen). Web monitoring is an ideal tool for this purpose because it enables the collection, storage, and analysis of real-world, detailed online activity relevant to cancer misinformation exposure. This methodology also advances previous research, which has primarily relied on aggregate digital trace data collected without participant awareness, by instead using a prospective, participant-consented approach.

## Purpose and Overview of the Original Study

The purpose of this viewpoint paper is to share lessons learned from this novel application of web-monitoring software for health communication research. We aim to provide practical information to other researchers who are exploring technology-based methods to objectively measure the quality of cancer information individuals are exposed to online.

This viewpoint paper reports lessons learned from a pilot study where we adapted commercially available web-monitoring software with the primary aim of describing the acceptability and feasibility as reported by participants. This prospective, sequential pilot study aimed to lay the groundwork for a scalable method to measure cancer misinformation exposure and its potential impacts on cancer outcomes. Participants completed a baseline survey, installed a commercially available web-monitoring software on their personal computer for 30 days, and then completed a follow-up survey. Survey data were collected and managed using REDCap electronic data capture tools hosted at University of Utah [15,16]. We recruited people from October 2022 to August 2023 with any cancer diagnosis who were aged 18 years or older, could read and understand English, and were willing to install the web-monitoring software on their personal device for the 30-day monitoring period. Participants did not need to be from the United States. In total, 56 participants completed the baseline survey. Of these 56 participants, many were unable to complete the web-monitoring period primarily due to technological reasons explained here as lessons learned. Ultimately, 19 participants attempted installation, with 17 successfully completing the software installation, 30-day web-monitoring period, and follow-up survey.

## Lessons Learned

The primary outcome of the pilot study was to evaluate the feasibility of adopting web-monitoring software to capture cancer-related web content (eg, search results and social media posts) in a research setting. In this paper, we use Proctor’s implementation outcomes (denoted here in italics) to explore the feasibility of this novel methodology and describe early-stage implementation lessons learned organized across three topics of (1) data quality (adoption and appropriateness), (2) software implementation (feasibility and fidelity), and (3) participant acceptability (acceptability) [17,18].

## Data Quality

Data quality was measured using implementation outcomes of adoption, or the intention to use an innovative tool (eg, web-monitoring software) and appropriateness, or the compatibility of the web-monitoring software to a research setting. Inherent to this goal were 2 priorities: (1) to maximize participant data security and (2) to maximize data quality of online cancer-related information. We assessed data quality primarily through consultation with IT subject matter experts and internal software testing.

To our knowledge, this is the first study adopting commercially available web-monitoring software for research purposes outside of opt-in panels offered by some survey vendors (eg, YouGov Pulse) [19,20]. Our first step of adoption was to obtain additional consultation from the University of Utah’s IT department to optimize and ensure participant privacy and security. A stipulation from the University of Utah IT specialists for the use of web-monitoring for research entailed establishing a partnership with the University’s Center for High Performance Computing (CHPC) to create a virtual machine (VM) to host all collected web-monitoring data. This was preferred over the standard cloud-based deployment of web-monitoring software as it allowed for the data to be kept on premise, stored on encrypted drives within CHPC’s Protected Environment, and data were also encrypted while in transit. The Protected Environment is maintained at a university-owned data center with restricted access and classified as Health Insurance Portability and Accountability Act (HIPAA) compliant (eg, specifically built for protected health information related research). Access to the VM itself was restricted to study staff who had been trained in responsible conduct of research and had specialized training in maintaining the privacy and security of data collected via web-monitoring. This specialized training was informal and occurred through regular small group instruction between the research staff and the technologists at the CHPC.

The next step was to select a web-monitoring software. Study staff first compared available features and data security measures of popular companies for alignment with study goals and security requirements. Then, we consulted with 3 companies that had unique features available for potential application for monitoring cancer misinformation exposure. Upon completion of these consultations, we selected the software that was the most compatible with our specific use case. In conjunction with engineers from the software company and CHPC staff, study staff [EW and CT] configured software settings to maximize monitoring of online cancer data and minimize unnecessary data collection, thus reducing participant risk and improving adoption. This was accomplished by (1) creating a custom dashboard on the internally hosted VM, (2) configuring settings to exclude potentially high-risk data sources (eg, banking sites, email, and medical records), and (3) using an “alert word” feature to target data collection around cancer topics. The alert word feature allows administrators to input a list of words that will automatically trigger a screenshot of the participant’s device. We input a list of 61 curated cancer terms from a published dictionary with words relating to cancer diagnosis and treatment as well as misinformation terms from our previous studies on lexical content of cancer misinformation (Multimedia Appendix 1) [6,21].

Once the software was installed on the participant’s computer, it appeared on an internally hosted dashboard accessible to restricted members of the study team. Study staff recorded the time stamp of the initial installation as the beginning of the 30-day web-monitoring period and instructed participants to engage in their regular web browsing activities. After installing the software, participants were told that if they planned to search for cancer information, they should use the computer equipped with the software during this 30-day period, as opposed to an unequipped cell phone. This limitation affected adoption and appropriateness, as other software companies may have better mobile device compatibility, which could make this methodology a better fit for measuring online misinformation.

We also assessed data quality through the lens of appropriateness, or the compatibility of this web-monitoring approach to effectively address a particular issue, as poor data quality would impact effectiveness. The internal dashboard used by the study team displayed data as they were collected from participant computers in real time, organized into the following sections: (1) activity log, which contained a list of websites visited; (2) search log, which contained text of web searches; and (3) screenshot log, which contained a screenshot and time stamp every time an alert word was viewed or typed. We found that this real-time, objective data collection of participant cancer-related web activity was highly appropriate for our research question.

However, we note here that the study team completed internal device testing and discovered a lack of sensitivity around accurate screenshot logging from social media platforms. This negatively impacted data quality, as social media is a critical source of data collection since a large amount of online misinformation appears on these sites [22]. Engineers at the software company were unable to determine the cause of this issue, and it may have resulted in missing data. To the best of the study team’s knowledge, the screenshot capture feature worked accurately on search engines and third-party websites (eg, not social media). This means that when a participant searched a cancer-related term on a search engine and clicked on a result leading them to a third-party website, the study team could see screenshots of the search results and subsequent webpage as well as time spent on each webpage in the activity log. The goal of this method is to improve on other digital trace data approaches, though digital trace data often struggles to capture social media data well. Improving on this specific aspect of our initial efforts would likely greatly improve data quality in the future and the validity of this approach.

**Figure 1.**
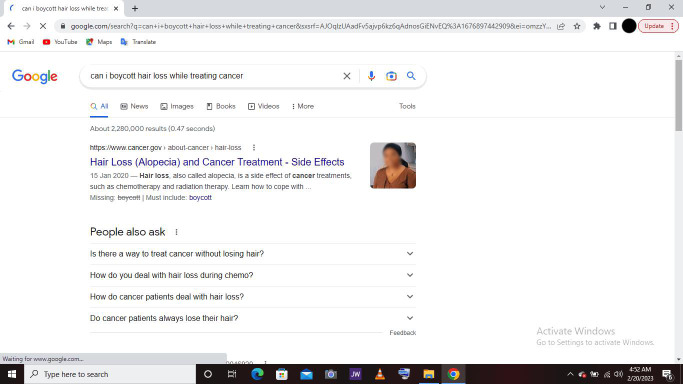
Screenshot of a participant’s computer screen captured by web-monitoring software.

Upon completion of the study, we had collected 11,978 entries in the activity log (mean entries per participant=448.88, range=0-5731), 2876 entries in the search log (mean=27.24, range=0-248), and 41 entries in the screenshot log (mean=2.41, range=0-15). Some specific participant web searches were about cancer cures (eg, “is there a permanent cure to cancer”), side effects (eg, “can i boycott hair loss while treating cancer”), symptom management (eg, “foods for cancer patients to eat”), and resources (eg, “is there any cancer support program in los angeles;” Figure 1). These data were collected from 17 participants who all completed the full 30-day monitoring period. Most entries in the activity log were appropriately cancer-related, indicating that the software was collecting data as intended. While some noncancer-related content was collected and displayed on the internal, password-protected dashboard, it was not maintained and was excluded from analysis.

## Software Implementation

Implementation of the web-monitoring software was assessed through 2 outcomes: feasibility, or the effectiveness of web-monitoring in capturing online cancer misinformation, and fidelity, or the degree to which study procedures aligned with the original protocol. We assessed these outcomes through 6 follow-up survey questions; 3 questions were related to fidelity and 3 were general feasibility questions (Table 1).

**Table 1.** Feasibility and acceptability questions.

Implementation outcome [17] and item	Response
Fidelity	
How helpful were the instructions emailed to you for the software installation? This includes the instructional video, written steps in the email, and links to additional resources.	Very helpful Somewhat helpful Neither helpful nor unhelpful Somewhat unhelpful Very unhelpful
Please share any additional feedback you have about the installation instructions.	Write-in box
Would you have preferred receiving a tablet pre-installed with the web-monitoring software and using it for any web-browsing related to cancer instead of installing the software on your own device? Please explain.	Yes – write-in box No – write-in box
Feasibility	
What device did you use for the web-monitoring period?	Windows computer Apple computer
Did you keep the web-monitoring software on your device for the full one-month period?	Yes No – Additional questions: How many days did you keep the web-monitoring software on your device? Please describe the reason you stopped using the software early.
Please share any additional feedback you have on using the web-monitoring software.	Write-in box
Acceptability	
How comfortable or uncomfortable did you feel with using the web-monitoring software?	Very comfortable Somewhat comfortable Neither comfortable nor uncomfortable Somewhat uncomfortable Very uncomfortable
How comfortable or uncomfortable did you feel with the web-monitoring software being on your devices?	Very comfortable Somewhat comfortable Neither comfortable nor uncomfortable Somewhat uncomfortable Very uncomfortable
Based on your experiences, would you recommend participating in this study, including agreeing to use the web-monitoring software, to other patients like you? Why or why not?	Yes – write-in box No – write-in box
Did you feel that the one-month web-monitoring period was:	Too brief About the right amount of time Too long

We assessed feasibility of implementing web-monitoring software from both study staff and participant perspectives, focusing on software installation of the web-monitoring software and the usability of the software throughout the 30-day web-monitoring period. Feasibility is distinct from appropriateness because while the web-monitoring software may be a good fit for measuring misinformation, it will not be a feasible intervention to scale up if the software is difficult to install and use. We found the installation process to be technically challenging (eg, requiring a high level of computer literacy) and resource-intensive (eg, requiring live IT staff videoconference assistance for 7 of the 19 participants who attempted the installation).

We provided participants 2 options to choose from when installing the software on their personal computers: (1) complete the installation independently by viewing an instructional video created by the study team and following along with written instructions or (2) schedule a Zoom (Zoom Communications) meeting with the study team and share their screen while they are talked through the installation steps in real time. We communicated to study participants that the installation process required intermediate computer literacy skills, and method 2 was recommended for most people. However, this design was intentionally chosen out of anticipation that participants would be less likely to participate if they were required to attend a meeting, which was confirmed when most participants initially opted to attempt the installation alone and others were lost to follow-up when they were unable to complete it this way. More participants completed the installation successfully independently than in a Zoom session, but it is unknown how long these participants had to spend on the installation and how many of the participants lost to follow-up at this stage gave up due to installation issues. Streamlining the software installation process and measuring implementation metrics such as time spent on installation should be a priority for future research using web monitoring approaches.

Of the 19 participants who attempted the installation, most participants (n=15) chose to complete the installation on their own and the remaining participants (n=4) chose to participate in a Zoom session. Of the participants who attempted the installation independently, 12 completed it successfully, 1 completed it after asking follow-up questions to the study team, and 2 needed to participate in a Zoom session to complete it with study team assistance. Of the 4 participants who opted for a Zoom session, 2 successfully completed the installation, while the other 2 were unable to proceed due to previously unknown antivirus software incompatibilities. Compatibility issues with mobile devices and specific antivirus software impacted fidelity, as IT staff had to deviate from protocol to troubleshoot with affected participants in the moment.

Participants were asked 2 feasibility questions related to implementation and usability of the software: (1) how helpful were the instructions for the installation and (2) would you prefer to use a tablet borrowed from the study team with the software preinstalled as opposed to your own device? Importantly, these questions were added mid-study after the unforeseen installation complications (eg, antivirus incompatibility) arose and were only asked to remaining participants (n=8). All those participants indicated that the software installation instructions made available to them (eg, an instructional email with written step-by-step instructions, an internally created video showing the installation steps with voiceover narration, and external links to the software company’s website) were somewhat or very helpful. However, 3 wrote that they still encountered issues with the installation; 2 of these participants needed to try the installation several times and 1 initiated a Zoom session with the study team to complete the installation. Of the 20 participants lost to follow-up after completing the baseline survey, it is unknown how many attempted the installation and dropped out of the study because of technological challenges.

Half of the participants (4/8, 50%) would have preferred receiving a tablet for the duration of the study with the software preinstalled, with write-in comments indicating the complicated installation process made this a preferable option. One participant also believed the tablet would have eased their discomfort with the security of the software being on their personal device. The other participants explained they would not prefer a separate tablet, as they believe it would be inconvenient to keep track of a separate device and try to remember to use it whenever they intended to search for cancer-related information. These participants acknowledge a noted limitation of this approach, which is the degree of artificiality in someone’s organic web-browsing and social media behaviors when asked to use a new, temporary device. Participants who still use their personal devices for web-browsing activities would generate an unknown amount of missing data that would be unmeasurable to the research team aside from participant self-reports.

Another software implementation issue inhibiting accurate data collection was the inability to install the software on certain mobile devices due to privacy settings enacted by hardware developers (ie, Apple and Android). Many (n=15) recruited participants declined because they claimed they only use their mobile devices to access social media and to search for information. While participants were asked to use the device installed with the software if they knew they would be searching for cancer information, it is possible they were exposed to cancer misinformation while using social media applications on their mobile devices, even if they were not intentionally searching for incorrect information [23]. These results present significant obstacles for scalability of future studies using this approach.

Given the limitations with the use of preinstalled tablets and the application of the commercially available software used in this pilot, future studies should continue innovating novel methods to collect reliable, objective online misinformation exposure data for research purposes. This study team has begun work on creating an internally developed browser extension containing web-monitoring features related to this specific use case (eg, alert word screenshots on search engines and social media sites only). Once developed, a tool of this nature could easily be adapted for different applications by customizing the alert word terms for topics other than cancer. Future work should also prioritize mobile device compatibility, as monitoring on mobile devices is commercially limited yet highly valuable for this research.

## Participant Acceptability

Acceptability was measured as the perception among participants that the web-monitoring methods were agreeable and that the study activities were satisfactory. We developed 4 questions related to acceptability (Table 1). The final domain of interest in this pilot study was participant acceptability of the web-monitoring software. Most participants who completed monitoring and the follow-up survey were somewhat or very comfortable with the general concept of using web-monitoring software (16/17, 94%), and 88% (15/17) were neutral or comfortable with having the software installed on their personal devices. One participant responded, “somewhat uncomfortable” to both questions indicated via write-in response that they are “not comfortable with giving over control of my computer to an outside source” and would have preferred to be given a tablet preinstalled with the software to use for the study. Participants who were comfortable with the software commented that they were already used to this type of software in their jobs and that they liked how they did not have to do anything with the software once it was installed. Out of 17, 2 participants (12%) believed the 30-day web-monitoring period was too long but did not elaborate.

Overall, the 16 participants out of 17 (94%) who expressed comfort with the software also indicated they would recommend this study and the use of web-monitoring for research purposes, indicating high acceptability of web-monitoring methods from the participants who completed the entire study. However, participants who consented self-selected and were undoubtedly predisposed to accept these methods since they learned about the web-monitoring software and agreed to install it on their personal device as part of the consent process. While a few people (n=4) who were approached about the study did decline upon learning about the web-monitoring, it is unknown how many potential participants passively declined due to their discomfort with the software or how many participants lost to follow-up dropped out because of a lack of acceptability. The inherent self-selection bias in this study design was a known limitation and was less of a concern for this pilot study as we were exploring the general use of this approach. However, our experiences mirror similar realities and blind spots that researchers regularly face when collecting and analyzing digital trace data [24].

Thus, assessing acceptability toward web-monitoring methods requires an understanding of individual attitudes and behaviors about personal data privacy, as feasibility will plummet if people are reticent about web-monitoring on principle. While data privacy attitudes can be largely heterogenous, a 2023 Pew survey found 83% of US adults with high cybersecurity knowledge reported they had taken at least 1 step online to remove or reduce their digital footprint, such as turning off cookies, using a private browser, or using a password manager, compared with 51% of adults with less cybersecurity knowledge [25]. This suggests many adults are taking at least some action to protect their data privacy, even if they lack specialized knowledge. Yet, another study from 2017 found that 89% of a 1500-person sample took at least 1 step to protect their data, but very few took all possible steps available to them [26]. This behavior may potentially be explained as “digital resignation,” a sense of helplessness in which an individual believes true safekeeping of their personal data is impossible in an increasingly digital world, and thus their response is a loss of vigilance that does not necessarily result from a lack of concern for their privacy [27]. Similarly, the 2023 Pew survey referenced above found that 74% of the adults with the highest cybersecurity knowledge who were taking the most actions to protect their data privacy also felt the most skeptical that “anything they do will make much difference” [25].

Researchers adopting web-monitoring methods should be aware of the highly complex and sometimes contradictory individual behaviors around data privacy (eg, feeling concerned about one’s digital footprint yet continuing to share information on social media for psychosocial benefits) and how that could impact research of this nature [26]. Specifically, this should include detailed consent documents that can be explained and understood in layman’s terms in an abbreviated manner to prevent the phenomenon of “uninformed consent” in which someone assumes a long and technically written privacy policy is protective and agrees without reading it. Second, researchers using these methods should use stringent protocols such as the ones outlined in this pilot study for storing, maintaining, deidentifying, restricting, and ultimately destroying web-monitoring data post analysis to mitigate as much risk as possible for participants.

## Additional Implications for Future Research

More work is needed to address technological and implementation limitations that hindered this pilot study. As researchers continue to explore this methodology, validated measures of implementation outcomes should be integrated to allow for a more objective understanding of how to optimize web-monitoring software for research purposes [28]. Proctor’s framework also includes 3 implementation outcomes not discussed here: cost, penetration, and sustainability. These additional outcomes should be formally measured and reported on in future scale-ups of this methodology.

Another important consideration for future research is data collection across different cancer types and time since diagnosis, as cancer is a heterogeneous disease and information-seeking needs vary across the cancer continuum. Information-seeking tends to be higher for patients recently diagnosed with or actively in treatment for cancer compared with other phases of the cancer continuum (eg, pretreatment, in-treatment, posttreatment, and recurrence) [29]. Online cancer misinformation exposure should be measured and compared between specific cancer types and time points since diagnosis, as exposure level and associated risk may vary significantly across these groups.

## Conclusions

Overall, our results indicate high acceptability among patients with cancer who consented to use web-monitoring software for research purposes. While we managed implementation issues related to ease of installation and sensitivity of the data collection, the software did succeed in capturing real-time screenshots from patients’ computers as they searched for cancer information online. This is a promising first step toward refining a tool of this nature to capture all cancer-related web content consistently and accurately, which will improve objectivity in this area of scholarship. Ultimately, these measurements are needed to better understand the scope of online cancer misinformation and the impact it may have on health outcomes for patients with cancer, a population navigating a complex disease in an increasingly complex online environment.

## Supplementary material

10.2196/65887 Multimedia Appendix 1 “Alert Word” term list.

## Abbreviations:

CHPC: Center for High Performance Computing
VM: virtual machine
HIPAA: Health Insurance Portability and Accountability Act
