# Supplementary material for: Evaluating the Feasibility of Web-Monitoring Methodology for Measuring Exposure to Online Cancer Misinformation
Source: JMIR Cancer. 2025 Jul 29;11:e65887. doi: 10.2196/65887 (PMC12306912; doi:10.2196/65887)
Supplement: Multimedia Appendix 1 [file cancer-v11-e65887-s001.docx]

**Multimedia Appendix 1: “Alert Word” term list**

| **Cancer Terms**^14^ | | | |
| --- | --- | --- | --- |
| malignan | myeloma | carcinoid | oligodendroglioma |
| carcinoma | neuroblastoma | lukemia | wilm's tumor |
| cancer | astrocytoma | germ cell tumor | clear cell |
| radiation therap | renal cell | desmoplastic | non-hodgkins lymphoma |
| radiotherap | squamous cell | wilms tumor | non hodgkins lymphoma neurblastoma |
| immuno therap | medulloblastoma | seminoma | neruoblastoma |
| immunotherap | adenocarcinoma | ependymoma | rhabdomyosaroma |
| chemo | retinoblastoma | thymoma | nuroblastoma |
| chemotherap | ductal carcinoma | nueroblastoma | nonhodgkins lymphoma |
| lymphoma | mastectomy | lumpectomy | rhabdomyosarcoma |
| sarcoma | neuroendocrine tumor | myloma | lung_cancer |
| melanoma | histiocytosis | langerhans | skin_cancer |
| glioblastoma | luekemia | ductile carcinoma | thyroid_cancer |
| **Additional Terms** | | | |
| alternative_cancer_treatment | cancer_prevention | cancer_treatment | natural_cancer_treatment |
| cancer_cure | cancer_side_effects | cancer_type |  |
